# Major Thrombotic Event Despite Anticoagulation in a Patient With COVID-19

**DOI:** 10.7759/cureus.8896

**Published:** 2020-06-29

**Authors:** Basma Ataallah, Aman Sharma, Sharmiz Tamanna, Joseph Ng, Gregory Haggerty

**Affiliations:** 1 Internal Medicine, Zucker School of Medicine at Mather, Port Jefferson, USA; 2 Internal Medicine, Northwell Health Mather Hospital, Port Jefferson, USA; 3 Pulmonary and Critical Care Medicine, Northwell Health Mather Hospital, Port Jefferson, USA; 4 Graduate Medical Education, Northwell Health Mather Hospital, Port Jefferson, USA

**Keywords:** covid-19, pulmonary embolism, thrombosis, therapeutic anticoagulation, coagulopathy

## Abstract

The outbreak of coronavirus disease 2019 (known as COVID-19), which started in Wuhan, China in December 2019, is caused by the severe acute respiratory syndrome coronavirus 2 (SARS-CoV-2). It has been associated with both venous and arterial thromboembolism likely secondary to significant cytokine activation and inflammation. Reports on the incidence of thrombotic complications, however, are not well documented. Our case will examine a young man diagnosed with COVID-19 who developed an acute, severe bilateral saddle pulmonary embolism while on prophylactic dose anticoagulation after being admitted to the hospital and treated for two weeks with significant improvement.

## Introduction

The novel coronavirus disease 2019 (COVID-19) is a viral illness caused by a virus belongs to a single-stranded ribonucleic acid (RNA) virus of the human coronavirus family, named for the crown-like protein spikes on their surface. Severe acute respiratory syndrome coronavirus 2 (SARS-CoV-2) transmitted via respiratory droplets is thought to attach to various cell types expressing angiotensin-converting enzyme 2 receptors (ACE2) on their surfaces like in lung alveolar cells, cardiac myocytes, the vascular endothelium, and other cells [1]. The proposed mechanism for thrombogenesis is that SARS-CoV 2 attaches to the endothelial cells which are known to express the ACE2 receptors. This attachment activates the primary coagulation pathway by inducing the endothelial cells to release the von Willebrand factor and factor VIII involved in platelet aggregating and clot formation [1,2]. This underlying process coupled with immobility, hypoxia, and acute inflammatory state can predispose the patients to developing deep venous thrombosis (DVT) and pulmonary embolism (PE) [1]. 

The presentation of this illness in patients admitted to the hospital varies significantly in severity. Patients may present with a number of complications, including arrhythmia, septic shock, acute respiratory distress syndrome (ARDS), and multiorgan failure. Due to elevated markers of coagulation identified in patients admitted with COVID-19, the mainstay of treatment is initiating therapeutic anticoagulation [1,3]. Therapeutic treatment, however, is not always sufficient in preventing adverse outcomes of thrombotic complications if not started earlier in the course of hospitalization as seen in this case report. 

## Case presentation

**Figure 1:**
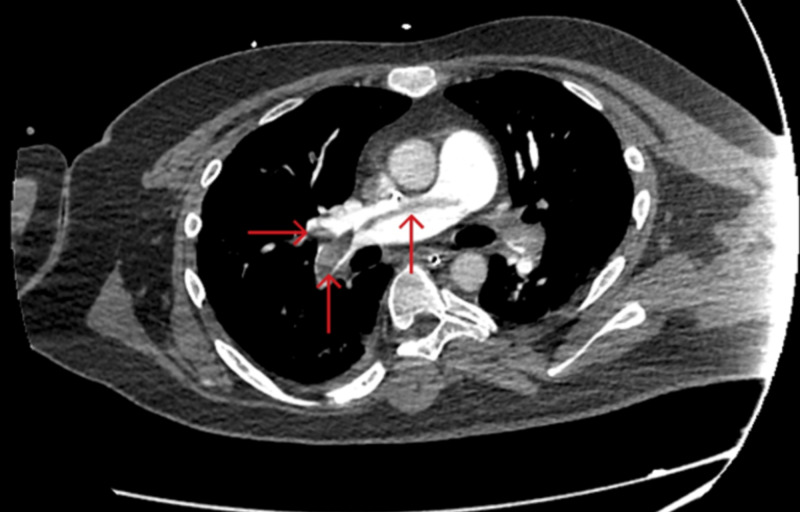
CT angiography of the chest with intravenous contrast shows saddle pulmonary embolism extending into bilateral main, lobar, segmental, and subsegmental pulmonary artery branches (red arrows), with CT features suggesting right heart strain.

Our patient is a 29-year-old man with mild, level 1 autism spectrum disorder, who was admitted to the hospital three days after the onset of fever and cough. He was initially treated with broad-spectrum antibiotics, as well as azithromycin and hydroxychloroquine for possible aspiration pneumonia and positive COVID-19 infection. On hospital day 12, the patient showed significant improvement of his overall symptoms. He was scheduled to be discharged in the next two days. That morning, however, a rapid response was called due to the development of acute, worsening dyspnea, and hypotension, requiring immediate intubation, mechanical ventilation, and transfer to the critical care unit. Norepinephrine and phenylephrine were added to his treatment as well due to worsening shock. The patient was restarted on broad-spectrum antibiotics and treated for septic shock and ARDS due to COVID-19 presumably complicated by a bacterial superinfection. During the course of his ICU stay, an elevated D-dimer level was observed at more than 5,000 ng/mL. The patient was switched from prophylactic anticoagulation to therapeutic anticoagulation regimen with a heparin drip due to concern for hypercoagulable state with COVID-19 on day 13. Imaging with CT angiography was performed due to further deterioration of respiratory status on day 14, which confirmed a bilateral saddle PE despite prophylactic anticoagulation (Figure 1). The patient was administered tissue plasminogen activator (tPA) and successfully extubated on day 18. He was transferred to the general medical floor on day 19. 

## Discussion

Recent studies have shown that elevated levels of coagulation factors are directly correlated with an overall worse prognosis and high likelihood of death in hospitalized patients. Elevated levels of D-dimer and fibrin degradation products have a roughly 3.5-fold increase in severe symptoms and death in COVID-19 infected patients [3]. A study from Icahn School of Medicine at Mount Sinai suggested that microthrombi, especially to the lungs, play a crucial role in mortality in patients with severe infection [4].

Preliminary data showed favorable outcomes and mortality benefit with therapeutic doses in hospitalized patient who had elevated levels of coagulation factors and D-dimers compared to those who received prophylactic doses. A case series examining a number of COVID-19 patients in the ICU setting with a remarkably high incidence (31%) of coagulation complications highlights the benefits of transitioning patients from prophylactic to therapeutic anticoagulation early on in their hospitalization [5]. Our patient’s hospitalization course coincides with the findings of these studies. The development of an acute, severe PE on prophylactic doses of anticoagulation calls for a much more vigilant approach to management of these patients. Venous thromboembolism (VTE) and PE need to be high on the differential diagnosis. Therapeutic anticoagulation needs to be started much earlier in the hospital course of COVID-19 infected patients especially when inflammatory and coagulation markers are elevated [5]. 

However, despite the new data showing high incidence of thromboembolic events in COVID-19 patients on therapeutic doses, there is still evidence of lower incidence of PE and VTE at therapeutic dose anticoagulation compared to prophylactic doses [6].

There continues to be a need for additional data for anticoagulation in COVID-19 patients. In the meantime, a careful risk stratification strategy can be implemented on case bases to prevent development of PE and VTE in this population even though recent studies have shown high incidence of thromboembolic events with therapeutic doses. 

## Conclusions

Due to the hypercoagulable state of COVID-19 infected patients, early management with anticoagulation is crucial for hospitalized patients in reducing mortality. Standard prophylactic anticoagulation is not sufficient in preventing thrombotic complications. Literature recommends switching standard prophylactic anticoagulation to therapeutic anticoagulation to mitigate microvascular and macrovascular complications. In patients who suddenly and unexpectedly deteriorate, clinical suspicion of a severe coagulopathic event should be high on the differential. This is clearly shown by the hospitalization course of our patient. As clinicians, we need to have a very low threshold when it comes to transitioning COVID-19 infected patients from prophylactic anticoagulation to full-dose therapeutic anticoagulation. 
